# The Hospital Saturday Fund

**Published:** 1887-04-30

**Authors:** 


### THE HOSPITAL SATURDAY FUND.

BY ONE OF THE MASSES.

Hospital Saturday.

"HOSPITAL SATURDAY" is a term with which the Londoner is now tolerably familiar. Once a year, the outsides of hospital walls and sundry other points of vantage are adorned by the bill-sticker with a bi-coloured poster, reminding us that Hospital Saturday is approaching. In due course Hospital Saturday comes. As busy City men hurry from their suburban homes to catch their trains and omnibuses, they encounter at station entrances, street corners, and other strategic points, neat little tables, on which are placed pretty little boxes in charge —generally speaking—of winsome maidens. We are reminded that "to give is a duty"; the hospitals are pleading; we must do our part to alleviate the ills and woes which environ our poor humanity. So we dive into our trousers' pockets, give our mite, and hasten on our journey.

Street and Workshop Collections.

This process of raising money for our charities, known as the "street collection," is all that a good many people know about Hospital Saturday; but it constitutes only one part, and that not the chief, and by no means the best, of the movement. The great metropolis is from one point of view a mighty hive of toilers. Workshops innumerable exist throughout London, giving employment to a vast number of skilled artisans. Here is a field, almost inexhaustible in extent, in which the promoters of the Hospital Saturday movement may labour with, I believe, the most successful results. Speaking of the work of the Fund in this direction, Lord Brabazon, in a letter to the Secretary, observes: "You have in the reserve subscribing powers of the workshops of London, now lying comparatively untouched, a future source of increased income to the hospitals, of which at present the public are, as a rule, utterly ignorant." This view I cordially endorse; indeed, after Mr. Alderman Spark's account of the Leeds system, the Council will be wise in abolishing the outdoor collections altogether.

What Last Year Accomplished.

Before going further, I will briefly state the practical result accomplished by the Fund last year. The total collection for 1886 amounted to £9,750, as against £9,500 in 1885. The day devoted to the collection last year, unfortunately, proved wet, and, in consequence, the street collection fell £500 below the anticipated amount; but the workshop collections showed a considerable increase, counterbalancing the street deficiency by £250. The collection was distributed among 132 institutions, namely, 75 hospitals and 57 dispensaries, convalescent homes, etc.

Far too Little.

Perhaps Mr. Frewer, the esteemed secretary of the Fund, will say I am unduly severe if I characterise as paltry the collection just completed, but it certainly seems to me that something must be radically wrong in a movement which has such extensive organisation, and such an immense field of exploration, when it produces a sum so wretchedly small. London and its suburbs have a population of something like five millions. Take a fifth of this number at a farthing a week, and we get a sum of £54,218. If a tithe of the workshops of the metropolis contributed each man one penny per week, the Hospital Saturday Fund would soon remove the pressing difficulties under which our charities labour.

A Peep at the Past.

The Fund was established in the year 1873, mainly through the exertions of Major (then Captain) Mercier, but the first collection was not made till October 17, 1874, when a trifle over £6,000 was raised. For the next six years a dead level was maintained. In 1880 the collection exceeded £6,600, and in the interim it has grown to the figure I have named. The Fund has an ample *raison d'être*. Before Hospital Sunday and Hospital Saturday were thought of, our charities, while sought after by the masses generally, had been mainly supported by the subscriptions of the wealthy few. Now, as long as this state of things could be made to last, there was perhaps no sufficient reason why those less happily circumstanced should be asked to give, but the time soon came when the cheques of the wealthy no longer sufficed to cover the expenditure entailed by the wants of the poor. Moreover, with modern science came an increase in the cost of management, and to the present hour this costliness has gone on increasing.

The Principle of the Fund.

Captain Mercier and those who co-operated with him in establishing the Hospital Saturday movement in London, were, in reality, only building on what had already been done by working men in Birmingham, Manchester, and elsewhere. The principle upon which they went was, that it was only just and right that the working-classes, who mostly received the benefits of our hospitals, should co-operate in support of their maintenance, instead of leaving the burden wholly on the shoulders of the wealthy. Of course, an objector could easily say, "Oh, there is no need for any organised medium; the artisan can give his pence to the hospital in the same way that the peer gives his pounds." This is perfectly true, if each mechanic would recognise a certain personal liability; but, unfortunately, the hard experience gained from facts teaches us that that which is everybody's business, in the end becomes no one's. To parody a sentence from Macaulay, "organisation is to charity, what steam to machinery, the grand propelling power." Where the masses have to be dealt with, nothing can be done without thorough organised effort. What the promoters of the Hospital Saturday Fund wanted to do was to enlist the sympathy and to secure the practical support of the men who, when they fell sick, came to the hospitals for relief.

A Reason for its Want of Progress.

The object was high-minded, noble, philanthropic. I am quite prepared to make ample allowances for the great difficulties which must inevitably be encountered in the inauguration of a movement so far-reaching in its ramifications as this. The scathing criticisms which were hurled against the council during the first few years of the establishment of the fund were perhaps scarcely justified. To spend in working expenses five or six shillings out of every twenty received, certainly betrayed something faulty in the organisation. I do not hesitate to say, that, in my opinion, the tardy progress of the Hospital Saturday movement has been in the main due to the inordinate expenses which have been incurred in the collection of the fund. I am glad, however, to be able to state that, during the past three years, the working expenses have been very considerably reduced. In 1883, they stood at nearly fourteen per cent., while last year, Mr. Frewer informs me, they were reduced to nearly ten per cent. But if the Hospital Saturday Fund is to live, rather than exist, if it is to gain the confidence of the artisan, I unhesitatingly say that the working expenses ought to be curtailed still further. With economy, I should think they might easily be brought down to from seven and a half to five per cent.

The Secretary of the Fund.

A few words about Mr. Robert Frewer himself. I never encountered a gentleman whose heart was more in his work than Mr. Frewer's is in his. He has been associated with the movement from the first. When the Fund was established, Mr. Frewer, who chanced to be a member of the Working Men's Club at Stratford, was nominated as Honorary Secretary for that district. For the succeeding five years he acted as Honorary Secretary to the Fund. In 1881 he received charge of the office, an appointment which, of course, necessitated his relinquishing his previous occupation, leaving him really in a worse position, pecuniarily speaking, than he was before. The change has proved of the utmost importance to the welfare of the Fund, and if Mr. Frewer does not weaken the force of his energetic action by having too many schemes on hand at once, he ought to bring the proceeds on Hospital Saturday to £20,000 in the present year. In any case, he deserves well of the public, and should be supported and encouraged in his work for the hospitals and the working-classes.

An Adjunct of the Fund.

Mr. Frewer has never lost an opportunity to increase the usefulness of the Fund. Indeed, I have sometimes been inclined to think that his energies have gone almost too far. He was mainly instrumental in bringing about the establishment of a convalescent home at St. Margaret's Bay, in connection with the Fund, and for which a separate collection is annually made. This excellent institution has now been established for three years, during which period nearly a thousand patients have been received. During the past year there has, I am pleased to learn, been a large increase in the number of ordinary subscriptions, and the Council have been enabled to pay off £750 of the debt, which now stands at £1,250. The Home, which contains twenty-four beds, was full during the greater part of last year, between five and six hundred men enjoying the advantage of a two to three weeks' stay.

Surgical Instruments for the Poor.

The provision of surgical appliances for the poor is another admirable scheme in connection with the Hospital Saturday movement. It was started just twelve months ago, and has been found to work well. Dr. F. Gordon Brown and Mr. S. H. Appleford have attended regularly every Thursday evening at the offices of the Fund, to see patients requiring instruments. These are obtained from the makers, the applicant paying half, or even less. During the year about 600 cases were relieved in this way, at a cost of about £300, of which about £100 was contributed by the patients themselves.

Consumption.

Mr. Frewer has been contemplating yet another "departure" from the original purpose of the movement, in the shape of a proposal for providing additional relief to consumptive patients. Consumption is at once one of the most distressing and most prevalent maladies which afflict the working-classes of this country. Mr. Frewer's scheme has been printed, and is to be laid before a Special Board meeting; and as there appear to be several points calling for discussion, it will, perhaps, be best now to refrain from entering into its details. It may, however, be said that his proposal seems a workable one, and, with some amendment, it will probably be found to supply a partial remedy for a grave want.

